# Reporting of statistical sample size calculations in publications of trials on age-related macular degeneration, glaucoma and cataract

**DOI:** 10.1371/journal.pone.0252640

**Published:** 2021-06-04

**Authors:** Sabrina Tulka, Stephanie Knippschild, Sina Funck, Isabelle Goetjes, Yasmin Uluk, Christine Baulig

**Affiliations:** Chair for Medical Biometry and Epidemiology (IMBE), Faculty of Health, Witten/Herdecke University, Witten, Germany; Universita degli Studi di Firenze, ITALY

## Abstract

**Background:**

Transparent and complete publications of randomised controlled trials (RCT) ought to comply with the guidelines of the CONSORT Statement, which stipulates sample size calculation as an important aspect of trial planning. The objective of this study was to analyse and compare the reporting of statistical sample size calculations in RCT papers on the treatment of age-related macular degeneration (AMD), glaucoma and cataract published in 2018.

**Material and methods:**

This study comprises a total of 113 RCT papers (RCT-P) published in 2018 (AMD: 14, glaucoma: 28, cataract: 71), in English or German, and identified through an internet-based literature search in PubMed and EMBASE. The primary outcome measure of the study was the number of trials providing a complete description of the underlying sample case calculation on the basis of the variables required (significance level, expected outcomes, power, and resulting sample size).

**Results:**

Of the RCTs reviewed, 64% (AMD), 61% (glaucoma) and 31% (cataract) provided a justification of the number of patients included. A complete description of the described studies’ sample size calculation including all the necessary values (primary outcome measure of this study) was described by 21% of the AMD, 29% of the cataract and 18% of the glaucoma RCT publications (in total: 24 of 113 (21%) at a confidence interval of 95%: [13%; 29%]).

**Conclusion:**

All three treatment areas analysed lacked reporting quality regarding the justification of the number of patients included in a clinical trial based on a sample size calculation required for ethical reasons. More than half of all RCT publications reviewed did not provide all of the required information on statistical sample size calculation, and thus lacked transparency and completeness. It is therefore urgently required to involve methodologists in a study’s planning and publishing processes to ensure that methodology descriptions are transparent and of high quality.

## Background

Randomised controlled trials (RCTs) are the gold standard in medical research [1] and therefore call for high quality in terms of content and methodology to meet this expectation. Correct trial planning requires statistical sample size calculation as sound justification for the number of patients to be included [2]. The sample size ought to be calculated very carefully for ethical and scientific reasons [3] in order to be able to substantiate actually existing treatment effects by conducting an RCT [4]. The quality of trials without statistical sample size calculation can be considered less high [5]. The ICH E9 Guideline on Statistical Principles for Clinical Trials in the USA, Europe and Japan requires in particular to perform, document and report statistical sample size planning, including all hypotheses and calculations [2].

Since 1996 (with revised editions in 2001 and 2010) the CONSORT (Consolidated Standards of Reporting Trials) Statement has supported authors by additionally supplying a checklist which specifies the details on trial planning, methods and results that are obligatory for a transparent, high-quality publication of trial results. An important aspect of trial planning listed in this checklist is statistical sample size calculation (item 7a). In addition, item 13a recommends graphical representation of the actual participant flow (i.e. the real number of participants) in a flow chart [1].

### Objective

Tulka et al. showed in a study comprising 97 RCT publications on AMD treatment published between 2004 and 2014 that only 18% of the publications reviewed provided a description of the sample size calculation [6, 7]. Charles et al. have already revealed a massive lack of reporting quality while showing that 43% of all RCT publications in six medical journals with the highest impact factor did not provide all the information needed for sample size calculation. Furthermore, they showed that about 30% of the recalculated sample sizes differed more than 10% from those published in the article [8]. Recently, Lee has shown that even in COVID-19 RCTs the reporting of sample size calculations was inadequate as only one out of four RCT publications reported a complete sample size calculation with a 6% difference between the reported and the recalculated sample size [9].

In addition to that, several other reviews [10–19] discovered a lack of quality in reporting sample size calculations in RCT publications. For example, a review by Lee and Tse showed that endorsing CONSORT by the publishing journal and a higher imapct factor in 2014 resulted in smaller differences between reported and recalculated sample sizes. But recalculation was only possible for less than half of the publications as only 40% provided sample size calculations with all the necessary elements (only 51.8% reported a sample size claculation). Furthermore, they revealed that the design of a study had an impact on sample size reporting as non-inferiority trials were better in reporting sample size calculations than studies with a crossover design [10]. Another analysis [11] showed that only 27.9% publications on phase 3 trials in oncology reported a complete sample size calculation with only 20.7% of these providing a rationale for the expected effect size of the primary outcome. Moreover, none of the analysed studies with a continuous outcome reported a complete sample size calculation. Chow et al. [12] found that not only the reporting of a sample size calculation was lacking but also the planning of a sample size calculation needs to be improved as they discovered that the majority of publications they reviewed expected larger effect sizes in the study planning as actually observed in the study results. These results were supported by another review in anaesthesiology which showed that over 90% of the studies provided at least one element of a sample size calculation but nearly one-third of the studies did not state a reason for the expected effect size, and in about one-fifth of the studies the expected effect size was missing [13]. Furthermore, appropriate reporting of sample sizes in publications on orthodontics was poor as less than one-third of the publications completely described the underlying sample size calculation. The reporting of complete sample size calculation was supported, however, when a statistician was involved in the study planning and the study was a multicentre trial [14]. The values of the expected elements also seemed to have an impact on the correctness of reporting [15]. Further systematic reviews proved that in addition to sample size calculation authors also avoid complete and sufficient descriptions of other methodological items of the CONSORT Statement, such as a definition of the primary and secondary study outcomes [16], as well as a figure illustrating the participant flow during the study (flow chart) [17]. Other aspects relating to sample size calculation should be improved in reporting too as, for example, RCTs were found that did not justify the sample size based on the primary endpoint [18] and did not report on how dropouts in the course of a study shall be handled [19].

Based on these findings, the objective of this study was to record the provision of complete descriptions of sample size calculations (descriptions containing the significance level α, the power (1 –β), the expected value for the primary endpoint for each group or an expected effect size, and the resulting target sample size) in publications of RCTs on the treatment of AMD, glaucoma and cataract within one year in order to compare the results obtained in these three treatment areas and to investigate whether factors promoting a (complete) description of sample size calculations can be identified.

## Material and methods

### Search strategy

Three PubMed^®^ and EMBASE^®^ searches for the investigated ophthalmic illnesses were performed to identify RCTs published in English or German between 1 January 2018 and 31 December 2018. Publications had to be designated as publications of RCTs on the treatment of AMD or cataract or glaucoma. Table 1 shows the complete search strategies in PubMed^®^ and EMBASE^®^. Any trials identified by EMBASE^®^ and PubMed^®^ were tested for eligibility (full text, description of RCT results) to ensure that only RCT publications were reviewed. A trial publication was considered eligible as suitable RCT publication if it provided information on a clearly randomised, prospective clinical trial on human participants with a suitable therapy of the respective ophthalmic illness. Publications that could not be clearly assigned to one of the indications were excluded (e.g. studies on the treatment of glaucoma AND cataract) in order to avoid any bias as a result of incorrect assignment. In the case of multiple publications only the first publication (the original publication of the registered study on the primary endpoint) was included. Any publications reporting secondary endpoints or secondary time points were excluded in order to avoid the evaluation of possible duplicate publications (publication bias).

**Table 1:** Search query.

**AMD**
**Search Query in PubMed:** (((macular degeneration) AND “”randomized controlled trial””[Publication Type])) AND (“”2018/01/01””[Date–Publication]: “”2018/12/31””[Date–Publication])”; Items found: 112, Time: 09:12:37
**Search Query in EMBASE**: (’macular degeneration’/exp OR ’macular degeneration’) AND [randomized controlled trial]/lim AND ([embase]/lim OR [medline]/lim) AND [2018–2018]/py
**Glaucoma**
**Search Query in PubMed:** (((glaucoma) AND “”randomized controlled trial””[Publication Type])) AND (“”2018/01/01””[Date–Publication]: “”2018/12/31””[Date–Publication])”; Items found: 79; Time: 09:17:29
**Search Query in EMBASE:** (’glaucoma’/exp OR ’glaucoma’ OR ’glaucoma surgery’/exp OR ’glaucoma surgery’) AND [randomized controlled trial]/lim AND ([embase]/lim OR [medline]/lim) AND [2018–2018]/py
**Cataract**
**Search Query in PubMed:** (((cataract) AND “”randomized controlled trial””[Publication Type])) AND (“”2018/01/01””[Date–Publication]: “”2018/12/31””[Date–Publication])”; Items found: 98; Time: 09:20:44
**Search Query in EMBASE:** (’cataract’/exp OR ’cataract’) AND [randomized controlled trial]/lim AND ([embase]/lim OR [medline]/lim) AND [2018–2018]/py

PubMed and EMBASE RCT search strategy for each of the investigated ophthalmic illnesses (AMD, cataract, glaucoma) in 2018.

**Fig 1:**
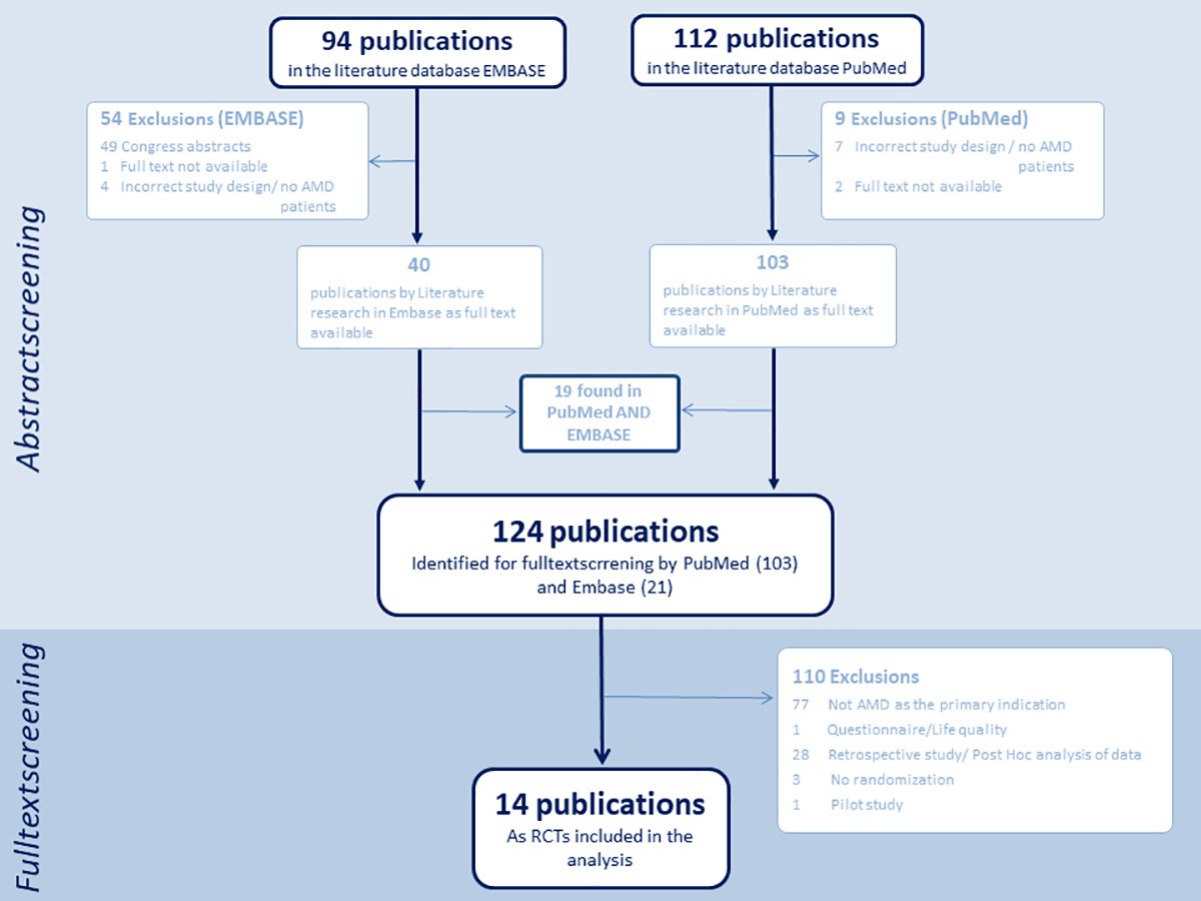
AMD flow chart. Flow chart on screening for publications of trials on AMD treatment.

**Fig 2:**
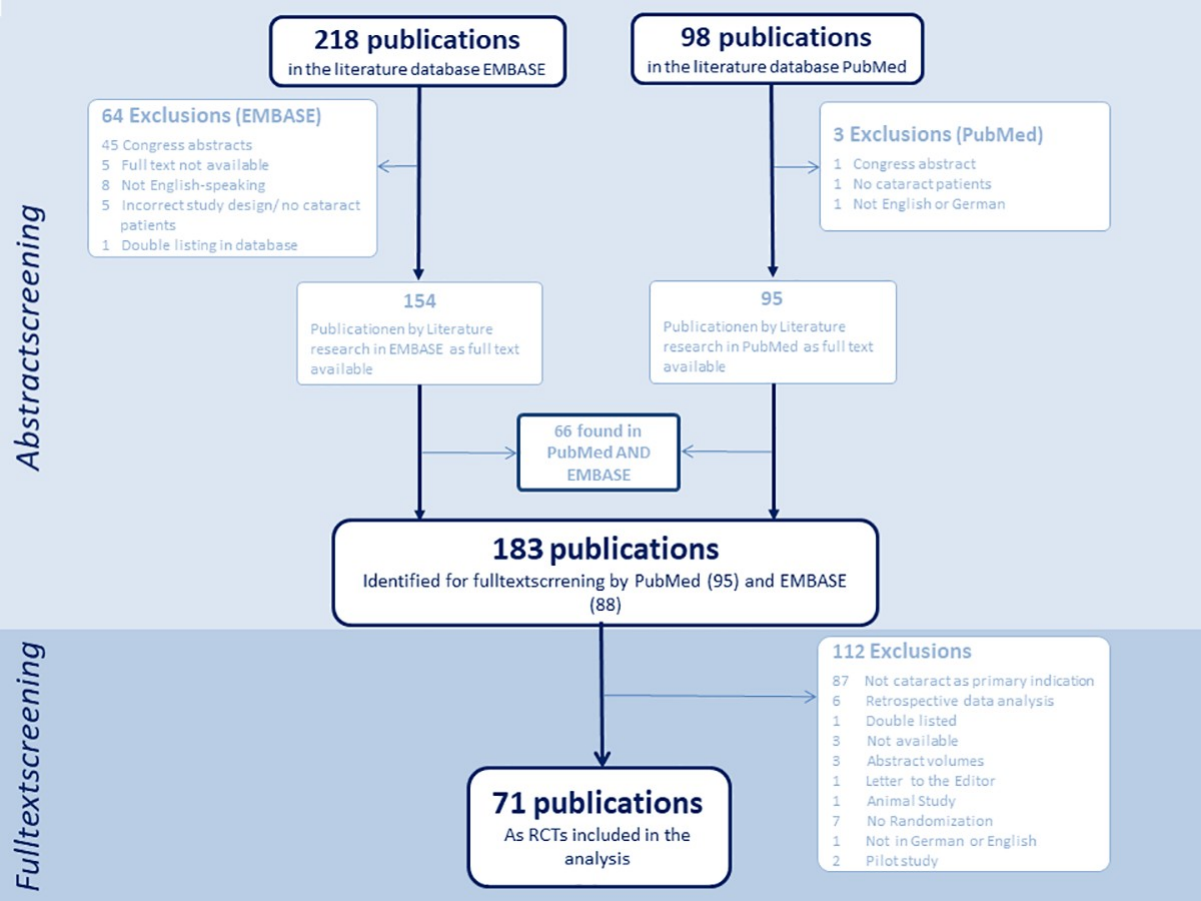
Cataract flow chart. Flow chart on screening for publications of trials on cataract treatment.

**Fig 3:**
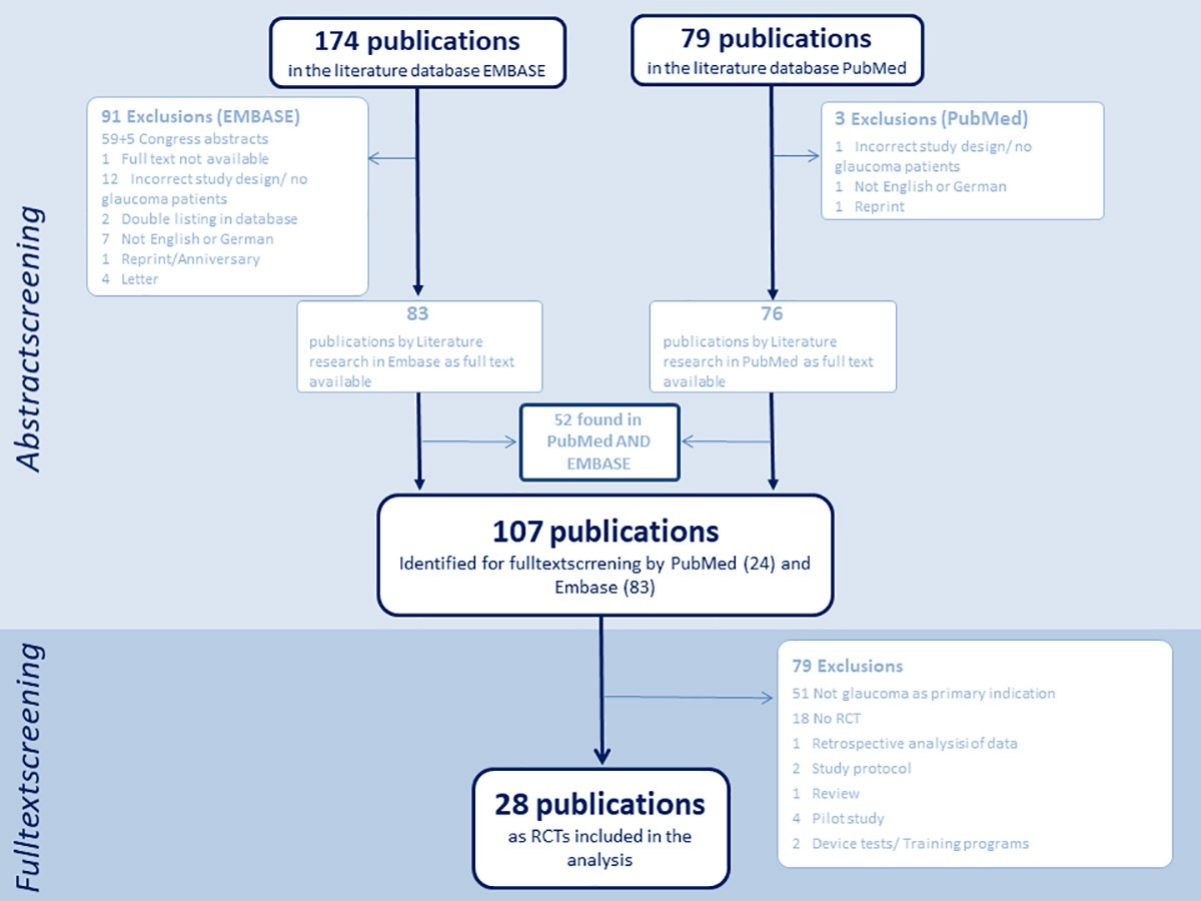
Glaucoma flow chart. Flow chart on screening for publications of trials on glaucoma treatment.

Furthermore, studies pooling multiple RCTs, protocols, pilot studies and conference abstracts were excluded. Fig 1–3 show flow charts of this verification process for each illness including specific exclusions.

### Data extraction

Publication data were extracted separately for each treatment area and collected in an Excel® spreadsheet, including title, authors, year of publication, the journal’s impact factor in 2018 and whether information on the sample size calculation was provided. One person each screened the publications to extract the data independently for each treatment area (AMD: SF, glaucoma: YU, cataract: IG). Afterwards, the data were checked for completeness and plausibility, and assessed by consensus rating (by CB, SK, ST). Data extraction comprised the treatment area, provision of a (complete) sample size calculation, presentation of flow charts, whether the trial was a multicentre trial and whether it had been registered on a trial registry, whether the journal referred to the CONSORT statement (listed as an endorser on: http://www.consort-statement.org/about-consort/endorsers1 (date: 2020/09/23)), the journal’s impact factor in the year of publication, number of groups, study design (parallel or crossover), allocation ratio and type of the primary endpoint (binary or continuous).

### Primary outcome measure and sample size calculation

The primary outcome measure of this study was the percentage of publications providing a complete description of the statistical sample size calculation (obligatory details according to CONSORT item 7a: significance level α, power (1 –β), expected value for the primary endpoint for each group or an expected effect size, resulting target sample size). In a primary analysis, the frequencies of reporting sample size calculations in publications of RCTs in the different treatment areas (AMD, glaucoma, cataract) were compared. As this study includes a review and complete data extraction of all RCT papers on the treatment areas published in 2018 (year of publication) was performed, statistical sample size calculation was not required for this study.

### Statistical analysis

Primary comparison and further secondary analyses were conducted descriptively by reporting absolute and relative frequencies and supplemented by 95% confidence intervals. In addition, logistic regression was performed for the primary outcome of the study and for the general reporting of a sample size calculation.

Based on the distribution of study characteristics (cf. Table 2), possible influencing factors were the treatment area (reference: AMD), the fact whether a trial was registered on a trial registry (reference: no), whether the trial reported was a multicentre trial (reference: no), whether the journal was one of the CONSORT endorsers, whether the journal had an impact factor in 2018 (reference: no) and the number of groups (two groups (reference) or more than two groups). Results were represented by the odds ratio and the related confidence interval of 95%.

**Table 2:** Study characteristics [a].

		All		AMD			Glaucoma			Cataract		
Characteristic		SSC no	SSC yes		SSC no	SSC yes		SSC no	SSC yes		SSC no	SSC yes
**CONSORT endorsed by journal** (n = 113)	*Yes*	11	8		0	3		1	2		10	3
	*No*	54	40		5	6		10	15		39	19
**Multicentre trial** (n = 113)	*Yes*	7	18		2	7		2	4		3	7
	*No*	58	30		3	2		9	13		46	15
**Registered on a trial registry** (n = 113)	*Yes*	19	30		1	7		7	13		11	10
	*No*	46	18		4	2		4	4		38	12
**Study design** (n = 113)	*Parallel*	62	44		5	8		10	15		47	21
	*Crossover*	3	4		0	1		1	2		2	1
**Allocation ratio** (n = 113)	*1*:*1*	46	40		4	6		8	17		34	17
	*Other*	19	8		1	3		3	0		15	5
**CONSORT flow chart published** (n = 113)	*Yes*	8	20		2	6		2	6		4	8
	*No*	57	28		3	3		9	11		45	14
**Primary endpoint** (n = 113)	*Binary*	5	6		1	2		3	3		1	1
	*Continuous*	11	39		3	6		1	14		7	19
	*No primary endpoint recorded*	49	3		1	1		7	0		41	2
**Number of groups** (n = 113)	*2*	53	39		5	8		9	16		39	15
	*3*	11	7		-	-		2	1		9	6
	*4*	1	2		0	1		-	-		1	1
**Impact factor in year of publication** (n = 113)	*Yes*	45	38		3	6		7	12		35	20
	*No*	20	10		2	3		4	5		14	2

Publication characteristics: CONSORT endorsed by journal, multicentre trial, registered RCT, study design, allocation ratio, published flow chart, primary endpoint, number of groups, impact factor in the year of publication associated with describing a sample size calculation (SSC).

Furthermore, complete descriptions of sample size calculations were recalculated with the data provided by the publications. All values used for recalculation were taken from the publication without changes. For these publications absolute and relative deviations between the recalculated and published sample sizes were determined (formulas: *p−b* and $\left(\frac{p-b}{b}\right)\cdot 100$, *with b = recalculated sample size, p = published sample size*). For publications with an allocation ratio that was not 1:1, the complete sample size was used instead of the sample size per group. Deviations and the distribution of the impact factor were described by minimum, median, maximum and quartiles as well as graphically with boxplots. Sample sizes were recalculated using the G*Power software and the R package TrialSize. R version 4.0.2 was used for data analysis.

## Results

### Publication characteristics

The number of RCT publications reviewed varied in the different treatment areas (AMD: 14, glaucoma: 28, cataract: 71). The following two tables (Tables 2 and 3) provide an overview of the factors that characterise the included study publications according to the description [a] and the complete description [b] of sample size calculations.

**Table 3:** Study characteristics [b].

		All		AMD		Glaucoma		Cataract	
Characteristic		CSSC no	CSSC yes	CSSC no	CSSC yes	CSSC no	CSSC yes	CSSC no	CSSC yes
**CONSORT endorsed by journal** (n = 113)	*Yes*	16	3	1	2	3	0	12	1
	*No*	73	21	10	1	17	8	46	12
**Multicentre trial** (n = 113)	*Yes*	17	8	6	3	5	1	6	4
	*No*	72	16	5	0	15	7	52	9
**Registered on a trial registry** (n = 113)	*Yes*	34	15	5	3	14	6	15	6
	*No*	55	9	6	0	6	2	43	7
**Study design** (n = 113)	*Parallel*	83	23	10	3	17	8	56	12
	*Crossover*	6	1	1	0	3	0	2	1
**Allocation ratio** (n = 113)	*1*:*1*	65	21	9	1	17	8	39	12
	*Other*	24	3	2	2	3	0	13	1
**CONSORT flow chart published** (n = 113)	*Yes*	21	7	6	2	7	1	8	4
	*No*	68	17	5	1	13	7	50	9
**Primary endpoint** (n = 113)	*Binary*	10	1	3	0	5	1	2	0
	*Continuous*	28	22	6	3	8	7	14	12
	*No primary endpoint recorded*	51	1	2	0	7	0	42	1
**Number of groups** (n = 113)	*2*	72	20	11	2	17	8	44	10
	*3*	16	2	-	-	3	0	13	2
	*4*	1	2	0	1	-	-	1	1
**Impact factor in year of publication** (n = 113)	*Yes*	65	18	7	2	14	5	44	11
	*No*	24	6	4	1	6	3	14	2

Publication characteristics: CONSORT endorsed by journal, multicentre trial, registered RCT, study design, allocation ratio, published flow chart, primary endpoint, number of groups, impact factor in the year of publication associated with describing a complete sample size calculation (CSSC).

Overall, only 49 of 113 RCT publications (8 out of 14 (AMD), 21 out of 71 (cataract) and 20 out of 28 (glaucoma)) stated that trials were listed on a registration platform for RCT trials. 46% (52 out of 113) of the RCT publications included did not define a primary endpoint for their study. Most of the publications that mentioned a primary endpoint were based on studies with a continuous outcome measure, and most of the publications described results of a study with two parallel groups of equal size. Eight AMD (57% of publications), eight glaucoma (29% of publications) and 12 cataract RCTs (18% of publications) included a CONSORT flow chart in their publication. Results of a multicentre trial were reported by 9 out of 14 (AMD), 10 out of 71 (cataract) and 6 out of 28 (glaucoma) publications. 83 of the analysed publications were published in a journal with an impact factor in 2018 (AMD: 9, glaucoma: 19, cataract: 55). The median impact factor in 2018 (year of publication) was 2.209 and ranged from 0.540 to 7.84 with 1.379 as the first and 3.085 as the third quartile. The complete distribution is shown in Table 4.

**Table 4:** Impact factor distribution.

	Minimum	1st quartile	Median	3rd quartile	Maximum
**All publications**	0.540	1.379	2.209	3.085	7.84
**AMD**	1.787	2.209	2.849	3.718	7.84
**Glaucoma**	0.540	1.349	1.787	3.072	7.84
**Cataract**	0.540	1.364	2.082	3.072	7.84

Distribution of the impact factors in the year of publication—2018 (in total and separately by treatment area).

### Primary outcome measure

Altogether, 48 of the 113 RCT publications reviewed provided a sample size calculation (42% of all publications reviewed; 95% confidence interval: [33%; 51%]) of which only 24 provided a complete description (primary endpoint of this study) including all details required by the CONSORT Statement that are necessary to enable replication (21% of all publications reviewed; 95% confidence interval: [13%; 29%]).

Table 5 shows that cataract had the smallest percentage of publications with (complete) sample size calculations. A (statistical) justification for the number of patients included was generally provided by 64% (AMD), 61% (glaucoma) and 31% (cataract) of the publications examined. A complete description of the sample size calculation was provided by 21% (n = 3) of the publications of trials on AMD treatment, by 18% (n = 13) on cataract treatment and by 29% (n = 8) on glaucoma treatment. Thus, a total of 33% (3 out of 9; AMD), 47% (8 out of 17; glaucoma) and 59% (13 out of 22; cataract) of the sample size calculations described were complete.

**Table 5:** Frequency of sample size calculations.

	Sample Size Calculation	Complete Sample Size Calculation
**All (n = 113)**	42% (95% CI: [33%; 51%])	21% (95% CI: [13%; 29%])
**AMD (n = 14)**	64% (95% CI: [39%; 89%])	21% (95% CI: [0%; 42%])
**Glaucoma (n = 28)**	61% (95% CI: [42%; 79%])	29% (95% CI: [12%; 46%])
**Cataract (n = 71)**	31% (95% CI: [20%; 42%])	18% (95% CI: [9%; 27%])

Percentage of sample size calculations and complete sample size calculations including 95% confidence intervals in total and separately by ophthalmic illness.

This shows that in 24 of the provided sample size calculations (AMD: 6, glaucoma: 9, cataract: 9) at least one of the required values was missing. Table 6 indicates how often each of the needed items (alpha, power, effect size, sample size per group) was reported in publications that mentioned a sample size calculation (n = 48). Nine out of the 17 publications without an expected effect size reported an incomplete effect (e.g. only the mean difference without the standard deviation).

**Table 6:** Missing values.

Value	Not reported
**Alpha**	2
**Power**	0
**Effect size**	17
**Sample size per group**	9

Frequencies of missing values in publications with reported sample size calculation.

### Logistic regression

Logistic regression for all trial publications analysed (n = 113) showed that a multicentre trial design, trial registration, glaucoma as the field of indication (compared to a trial on AMD), a group size larger than two and a higher impact factor had a positive effect (with an odds ratio larger than 1) on both the general reporting of the sample size calculation and on the completeness of the justification of this sample size calculation (primary outcome). In addition to that, a study on cataract had a positive effect on the completeness of a sample size calculation (as compared to AMD). Endorsing the CONSORT Statement by a journal had a negative impact on the general and the complete reporting of sample size calculations. The multicentre study design had a local statistically significant impact on the general reporting of sample size calculations (cf. Table 7) at a significance level of 5%. The remaining factors did not have a statistically significant impact on both the general and the complete reporting of sample size calculations in RCT publications.

**Table 7:** Logistic regression on reporting of sample size calculation.

Variable	Odds ratio	Confidence interval of 95%
**Outcome variable: sample size calculation**		
Intercept	0.31	[0.04; 2.26]
Registered (reference: no)	2.80	[1.10; 7.17]
Multicentre trial design (reference: no)	3.46	[1.13; 10.65]
Glaucoma	1.11	[0.23; 5.23]
Cataract	0.45	[0.11; 1.89]
CONSORT endorsed by journal	0.65	[0.19; 2.17]
Group size (reference: two)	1.00	[0.33; 3.07]
Impact factor (reference: no)	2.33	[0.84; 6.52]
**Outcome variable: complete sample size calculation**		
Intercept	0.07	[0.01; 0.71]
Registered (reference: no)	2.65	[0.92; 7.61]
Multicentre trial design (reference: no)	1.99	[0.62; 6.36]
Glaucoma	1.68	[0.31; 9.12]
Cataract	1.53	[0.30; 7.92]
CONSORT endorsed by journal	0.48	[0.12; 1.94]
Group size (reference: two)	1.34	[0.37; 4.89]
Impact factor (reference: no)	1.19	[0.40; 3.53]

Results of logistic regression (top: description of sample size calculation; bottom: complete description of sample size calculation), including influencing factors, odds ratio and confidence interval of 95%.

### Recalculations of reported sample size calculations

Recalculations of reported sample size calculations based on the variables stated in the publication resulted in per cent deviations from -93.55% (recalculated sample size: n = 827; published sample size: n = 57) to 107% (recalculated sample size: n = 39; published sample size: n = 80) with a median deviation of -2.4%. Table 8 shows the distribution of deviations for all publications that provided enough information on how the sample size was determined and separately for each treatment area.

**Table 8:** Distribution of deviations.

	Minimum	1st quartile	Median	3rd quartile	Maximum
**All publications**	-93.55%	-19.15%	-2.40%	0.13%	107%
**AMD**	-32.14%	-15.57%	1.01%	53.95%	107%
**Glaucoma**	-51.11%	-3.97%	-1.25%	3.85%	85.71%
**Cataract**	-93.55%	-51.11%	-3.53%	-1.39%	11.11%

Distribution of per cent deviations between recalculated and published sample size (in total and separately by treatment area).

**Fig 4:**
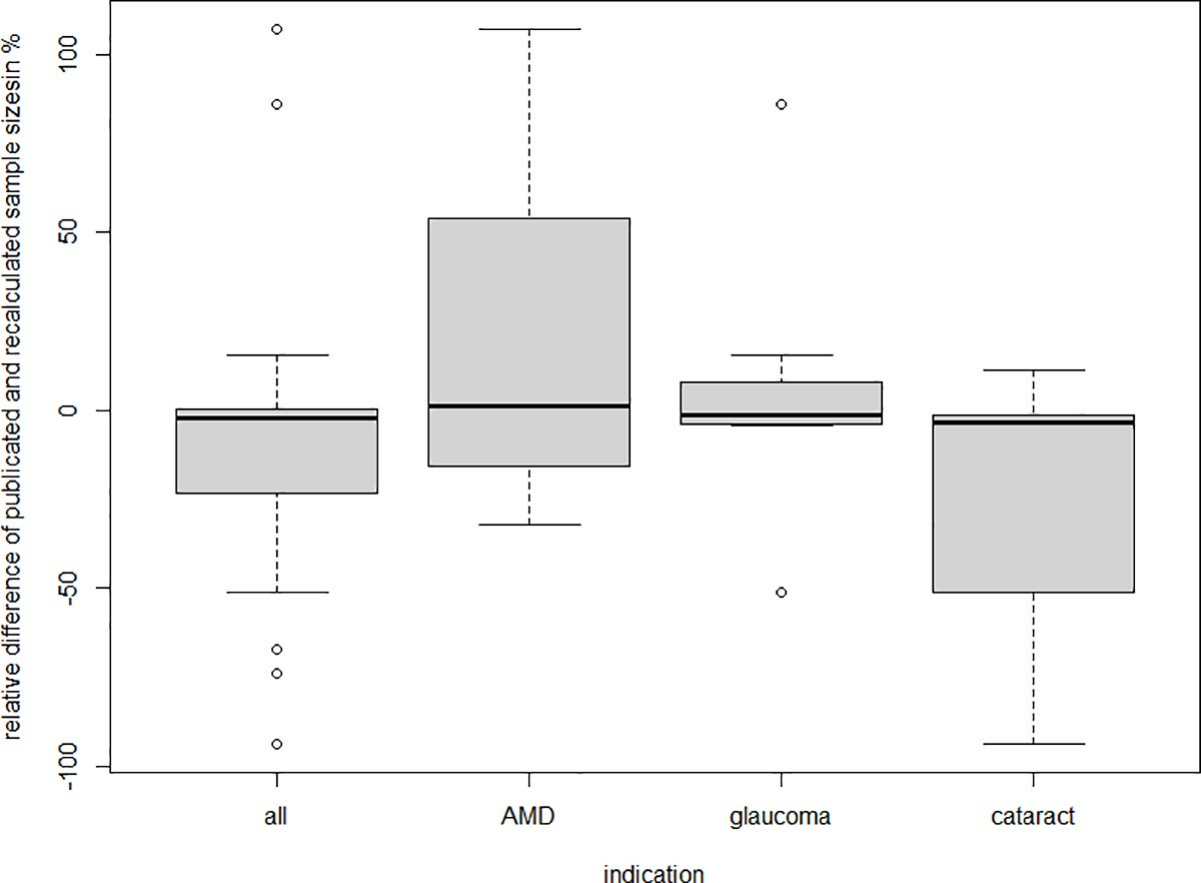
Per cent deviation boxplot. Boxplot on the distribution of per cent deviations between recalculated and published sample size (in total and separately by treatment area).

Fig 4 illustrates the distributions in a boxplot for all publications with recalculation of sample size and for each of the indications.

Altogether, 11 out of the 24 recalculated sample size calculations showed deviations of less than ± 5%, and 12 of the 24 publications showed deviations between the reported and the recalculated sample size of more than ± 10%.

## Discussion

The CONSORT Statement stipulates the provision of a complete description of the statistical sample size calculation as well as the representation of the actual patient flow in a flow chart for each RCT publication to ensure that these publications are transparent and comprehensible. This study analysed 14 RCT papers on age-related macular degeneration, 28 on glaucoma and 71 on cataract published in 2018 for a complete description of the statistical sample size calculation (including significance level, power, estimated outcomes and resulting target sample size).

Only 64% of the AMD papers, 61% of the glaucoma papers and just 31% of the RCT papers on cataracts published in 2018 justified the number of patients included in the trial at all.

The percentage of all trial publications in a treatment area providing a complete description of the sample size calculation was even less. Significantly less than half of all RCT publications reviewed in each treatment area provided complete and thus transparent and well-founded sample size calculations to justify the number of patients willing to participate in the trial. Moreover, recalculating the variables reported by complete descriptions of sample size calculations resulted in significant deviations (over- and underestimations) between the variables published and the sample sizes recalculated on the basis of these variables. Less than one fifth of all publications of trials in the ophthalmological treatment areas examined provided flow charts with information on patient trajectories in the course of the trial and, in particular, on a sound justification for early trial discontinuation. It is therefore more difficult for readers and especially clinicians, who need to take therapeutic decisions on the basis of such non-transparent publications, to interpret the trial results correctly. Reasons for the absence of relevant quality aspects of trials (or trial publications) are unknown and can only be guessed.

Endorsing the CONSORT Statement by a did not seem to increase the general and the complete reporting of sample size calculations. Only 17% (3 out of 18) of the articles published in a CONSORT-endorsing journal reported a complete sample size calculation. This fact is very surprising as the CONSORT Statement calls for a complete and transparent sample size calculation. Maybe completed CONSORT checklists that have to be uploaded when transmitting a manuscript were not checked or only checked superficially for correctness and completeness by the editors or reviewers. Reasons for this effect are unknown, however, and can only be assumed. Further studies may investigate this fact more deeply.

### Limitations

The verification whether publications found in PubMed^®^ and EMBASE^®^ were RCT publications being suitable regarding the treatment area in question was not performed by the same person for all trial publications reviewed and included in this analysis but by a single reviewer for each treatment area. In order to avoid a bias these reviewers followed a structured guidance that was developed prior to the start of this examination and checked for suitability by application in further quality checks for other indication areas.

Restricting the publication period to only one year (2018) is a further limitation of this study. This is related to resource constraints as the work presented is in an initial research phase. Moreover, this investigation was carried out as a project work by students of human medicine at the UWH and was carried out as a pilot project for a further larger investigation.

The resulting study’s limited sample size, especially for each type of research field, is the main limitation of this work. Therefore, a broader review covering a longer publication period is currently being performed.

The CONSORT Statement serving as the basis of this study was first published in 1996 and the version used for this study in 2010. For this reason, the period of time between the first availability of the CONSORT Statement and the publication period reviewed is considered long enough for authors to be able to report the content and information stipulated by the Statement completely and correctly in all RCT publications. This study indicated that a journal’s reference to CONSORT 2010might not necessarily result in a complete description of sample size calculation as only a few journals were asking for compliance. This could be explained by the limited sample size of this work. Additionally, as the implementation of CONSORT is part of the gold standard for compiling a paper it can be expected that authors use the CONSORT checklist as a guideline for writing their papers.

The software originally used for sample size calculations was not necessarily also used for the recalculations as the software was not mentioned in the publications, which may have explained minor deviations. The major deviations often observed, however, cannot be attributed to the software and are therefore inexplicable.

If an effect size was directly reported we exactly used the reported effect size for calculation. If only the values for calculation were reported (e.g. mean and standard deviation for continuous outcomes or frequencies in the case that the reported outcome was binary) the software for sample size calculation directly used these values for calculation of the expected effect size. In these cases the standard formulas for effect size calculation were used. It was expected that authors that provided complete sample size calculations would have provided the calculated effect size if non standard formulas were used. Therefore it was assumed that a potential miscalculation of the effect size used negligibly influenced the recalculation of sample sizes. However, a certain among of bias can not be completely ruled out.

### Literature discussion

To the best of the authors’ knowledge, no study has been conducted so far comparing the reporting of sample size calculations in publications of RCTs on the treatment of AMD, glaucoma and cataract in 2018. Nevertheless, the description of sample size calculations in RCT publications has indeed been investigated before.

#### Completeness of sample size calculations

Regarding the treatment of AMD, a study by Tulka et al. [6] examined the completeness of the descriptions of sample size calculations in RCTs published between 2004 and 2014. It showed that only 18% (17 out of 97) of the trial publications provided all required sample size calculation variables. Compared to the frequencies determined by the former study, rates have little improved in the AMD treatment area (now 21% instead of 18%). This percentage, however, continues to be small, which also applies to the other treatment areas examined. The share of 18% in RCTs on cataract treatment in 2018, in particular, is similar to the frequency found in RCTs on AMD treatment published between 2004 and 2014.

A study by Charles et al. (2009) on descriptions of sample size calculations in RCTs from six journals with a high impact factor published between 2005 and 2006 showed that 43% of the 215 publications provided a complete description of the sample size calculation [8], independent of the underlying treatment area. Thus, their study revealed a similar trend as the one by Tulka et al. (2019).

Even Lee (2020) demonstrated that even in the new research field of COVID-19 a lack of reporting quality of sample size calculations existed. Only one of four (25%) publications of RCTs on COVID-19 patients published between 1 January 2020 and 4 April 2020 reported a complete sample size calculation that offered the chance to replicate the published sample size needed. This is comparable to the frequency of 21% of complete sample size calculations found for the ophthalmic RCT publications reviewed in this article [9].

Lee and Tse (2017) found a gap in documentation in publications from 2014 as that only 51.8% of all RCT publications indexed in PubMed and published in December 2014 included a sample size calculation while only 40% offered a complete description of the underlying sample size calculation [10].

A review on 140 publications on phase 3 trials published between January 2008 and October 2011 in one of six journals (Annals of Oncology, Cancer, Journal of Clinical Oncology, Journal of the National Cancer Institute, Lancet Oncology, The New England Journal of Medicine) illustrates that only 27.9% of all the publications completely described the sample size calculation (similar to our result of 21% of complete sample size calculations). The authors found that 93.6% of the studies reported the desired level of significance whereas 10% did not report whether it was one- or two-sided. The power was reported in 90.7% and the expected difference in 88.6% of the publications, but in only 57.9% of the reports the expected effect in the control and the intervention group, respectively, was mentioned [11].

Publications of anaesthesiology RCTs provided complete descriptions of sample size calculations with 66% [12] or 80.3% [13] (of the publications that provided a sample size calculation) more often than RCTs in this study on ophthalmology. For example, Chow et al. reviewed 255 publications from superiority RCTs with two parallel groups from six anaesthesiology journals (Anaesthesia, Anesthesia & Analgesia, Anesthesiology, British Journal of Anaesthesia, Canadian Journal of Anesthesia, European Journal of Anaesthesiology) in order to find out whether complete descriptions of sample size calculations were provided [12].

A review on 194 parallel group superiority RCTs in anaesthesiology (published in 2013) reported that 91.7% of the analysed publications included at least one value needed for sample size calculation and 73.3% of all publications reported all elements required [13].

The frequencies observed in ophthalmology can be compared with 29.3% in dentistry [14] and 29.5% in orthodontics [15]. The first review on 413 publications in orthodontics [14] published in six journals (American Journal of Orthodontics and Dentofacial Orthopedics, British Journal of Oral and Maxillofacial Surgery, International Journal of Prosthodontics, Journal of Clinical Periodontology, Journal of Endodontics, Pediatric Dentistry, Journal of the American Dental Association, Journal of Dental Research) between 1992 and 2012 documented that of these publications 121 offered a complete sample size claculation [14].

A review on 139 RCTs from eight journals (American Journal of Orthodontics and Dentofacial Orthopedics, Angle Orthodontics, European Journal of Orthodontics, Orthodontics and Craniofacial Research, Australian Orthodontic Journal, Journal of Orofacial, Orthopedics, Art and Science of Orthodontics and Dentofacial Enhancement (formerly World Journal of Orthodontics), Journal of Orthodontics) in the field of orthodontics showed that only 41 RCTs offered enough information to enable recalculation for readers of the study reports [15].

Zhang et al. did not find any publication providing appropriate information on sample size calculation (power, significance level and sample size) when reviewing 222 RCTs published in seven orthopaedic journals from China (Chinese Journal of Osteoporosis, Chinese Journal of Spine and Spinal Cord, Orthopedic Journal of China, Chinese Journal of Orthopaedic Trauma, Chinese Journal of Trauma, Chinese Journal of Orthopaedics, Chinese Journal of Hand Surgery) between 2010 and 2014 [19].

#### Recalculation of sample size calculations

Recalculation of the reported variables in publications on AMD revealed that only 8% (8 out of 97) of the publications provided an accurate description of the sample size calculation with a maximum difference between recalculated and reported sample size of 2 [6]. In the context of this study, recalculations identified an absolute maximum deviation of two individuals per group only in 10 out of 24 sample size calculations (42% of the complete sample size calculations were thus considered accurate, i.e. 1 out of 3 AMD trial publications). This corresponds to 9% of the publications reviewed (as per treatment area: AMD 14%, glaucoma 18% and cataract 8% of all RCT publications).

A study by Charles et al. (2009) showed a difference between reported and recalculated sample size greater than 10% in 30% (47 out of 157) of the articles with enough information for recalculation) [8].

In the new research field of COVID-19 replication of sample size calculations with the reported values led to a sample size that was 6% higher than the one reported in the analysed publication [9].

40% of all RCT publications that were published in December 2014 and could be found in offered enough information for recalculation with a median difference between reported and recalculated sample size of 0% (interquartile range: -4.6% to 3%). Recalculation of the sample sizes on the registry led to larger differences between sample sizes than for the sample sizes reported in the publication (median difference between registered and reported sample sizes: 0.3% with an interquartile range from -8.1% to 15.1%). Only 39.7% of the studies that reported a target sample size in both (registration and publication) also reported the same sample sizes in both [10].

A replication of sample size calculations in publications of RCTs in anaesthesiology showed that 70% of the publications with complete descriptions had differences between reported and recalculated sample size calculations of less than 10%; 51% of the analysed publications included a complete sample size calculation) [12].

In another review of RCT publications in anaesthesiology the replication of the complete sample size calculations showed differences for 32% of the publications, with 29% of the publications showing a difference of more than 10% [13].

121 orthodontic publications that had enough information for replicating the reported sample size led to median differences between the reported and the replicated sample sizes of 15.2% with a range from -237.5% to 84.2% [14].

In a second review on 139 RCTs in orthodontics a median difference of 5.3% with a range from -93.3% to 60.6% resulted from the authors’ recalculations [14].

#### Impact on sample size calculation

Our study shows that a higher impact factor increased the chance of describing a (complete) sample size calculation in an RCT publication on AMD, glaucoma or cataract. Another study by Tulka et al. (2020) supports these findings too, as they demonstrated that the frequency of sample size calculation descriptions was higher if the impact factor of the publishing journal was higher [7].

Lee and Tse found in their linear regression that a higher impact factor and publishing in a journal that referred to CONSORT reduced the deviation between the reported and the recalculated sample size. Additionally, they discovered that only 42.8% of the reported trials were registered on a trial registry (50.8% of these with a sample size calculation in the registration). Furthermore, the authors showed that the study design had an impact on the reporting of sample size calculations as 80% of all non-inferiority trials and only 22.6% of all studies with a crossover design reported complete descriptions of how the sample size was calculated [10].

This analysis partly differs from the results of our binary logistic regression on the reporting of all values needed to calculate a sample size (yes or no) that showed that endorsing the CONSORT Statement did not increase the chance of describing a complete sample size calculation in a study’s publication. However, the positive influence of a journal’s impact factor (across indications) could also be demonstrated in this investigation (for the ophthalmic RCT publications).

Another study on 140 publications on phase 3 trials published between January 2008 and October 2011 in one of six journals analysis revealed that 42.9% of the studies with a categorical endpoint, 23.3% of the studies with a time-to-event endpoint and 0% of the studies with a continuous endpoint published a sample size calculation with all the values required. Our conclusions are different as in our study only 9% of the publications with a categorical primary outcome but 44% of the studies with a continuous primary outcome reported a complete sample size calculation. Besides, 46% of the publications analysed in this study did not define a primary endpoint [11].

A logistic regression in orthodontic publications revealed that the involvement of a methodologist improved the reporting quality of sample size calculations. The same trend was shown for publications reporting a multicentre trial and publications with a later year of publication [14].

Koletsi et al. found that the size of the differences seemed to be influenced by the value of the power used, as publications with a power of 80% had a median difference of 5.3% while the differences were 45% and -30% for publications reporting a power of 85% and 90% respectively [15].

Zhang et al. revealed that another important aspect associated with sample size calculation was inadequately reported as only 7.2% of the publications analysed included a description of how dropouts should be handled [19].

Compared to Tulka et al. (2019), our review of later publications showed a slight improvement in the field of AMD treatment.

Chow et al. [12] were also able to demonstrate these changes in trend over time with a frequency of complete descriptions of sample size calculations of 56% in 2010 and 84% in 2016 in publications of RCTs in anaesthesiology.

In dentistry [14], too, it was possible to prove these changes in trend over time by multivariate analysis, indicating that a later year of publication had a positive impact on the description of sample size calculation provided in a publication.

#### Differences in effect sizes

Chow et al. discovered that the expected effect sizes in the sample size calculations often did not match the reported results (for binary outcomes: 68% with expected effects larger than reported; for continuous outcomes: 56% with expected effects larger than reported) [12].

Additionally, Abdulatif et al. showed that in 19.7% of the studies on anaesthesiology the expected effect size was the element missing for replication, and of the publications reporting the expected effect size, 32.2% did not justify the anticipated values for the primary outcome measure [13].

#### Compliance with CONSORT

Huang et al. checked 182 RCTs in otorhinolaryngology from 10 top journals in the field of otolaryngology–head and neck surgery, published between 2010 and 2014, for compliance with the CONSORT 2010 Statement. They found that the analysed publications included between 25% and 93.5% of the items required by CONSORT (median: 59.4%). Sample size calculation was one of the aspects mentioned by CONSORT that was reported by less than 50% of the RCTs (40.6%). Other important items that were inadequately reported were the outcomes (primary and secondary) with 42.3% and the effect size with 32.4%. Our review showed that 47% of the AMD publications did not define a primary endpoint [16].

In addition to that, a study on reporting quality that investigated compliance with CONSORT in 185 RCT publications on dental bleaching published between 1996 and 2016 checked whether 16 items were reported and whether the descriptions were sufficient. They found an average reporting of 52.2% of CONSORT items while the sample size calculation and flow chart were not included in more than 80% of the reports. Less than 20% of the publications described the sample size calculation adequately and included a flow chart as required by the CONSORT Group [17].

Satpute et al. (2016) analysed the reporting of CONSORT items 7a (sample size calculation) and 12a (primary and secondary outcomes) in 174 RCT publications released in five pharmacological journals (The Journal of Clinical Pharmacology, British Journal of Clinical Pharmacology, European Journal of Clinical Pharmacology, Journal of Pharmacology & Pharmacotherapeutics, Indian Journal of Clinical Pharmacology). Only 59.2% of the publications stated the methods used for the sample size calculations, of which only 72.8% were based on the study’s primary endpoint (others were based on previous studies (21.4%) or feasibility (5.8%)). The authors did not investigate whether sample size calculations were reported completely but showed that only 40% of the publications mentioned the level of significance and power [18].

## Conclusions and prospect

Statistical sample size calculation has to be performed for each RCT to ensure the validity of results and is therefore explicitly stipulated by the ICH E9 Guideline for each clinical trial [2]. Nevertheless, the publication quality regarding this important aspect of trial planning was rather low in the fields of ophthalmology examined, as far more than half of all RCT publications reviewed did not contain all the information on statistical sample size calculation required. This implies that (statistical) methodologists should by all means be involved in each trial’s planning and publication processes to ensure transparent, high-quality methodology descriptions. Furthermore, it is essential that editors and reviewers always check trial publications for descriptions of statistical sample size calculations. If these descriptions are missing, each reviewer should insist on the provision of detailed information on sample size calculations.

## Supporting information

S1 Table Sample sizes. Recalculated and published sample sizes, difference between recalculated and published sample size, as well as per cent deviations between recalculated and published sample size.
(DOCX)

S2 Table Study characteristics. Publication characteristics: CONSORT endorsed by journal, multicentre trial, registered RCT, study design, allocation ratio, published flow chart, primary endpoint, number of groups, impact factor in the year of publication.
(DOCX)

S3 Table Deviations of sample sizes. Per cent deviations between recalculated and published sample size using the formula (p–b) / b as compared to (b–p) / b.
(DOCX)

S1 File Data set. Complete data set with all publications analysed and all data recorded including the following variables: Study, indication, FP, alpha, power, effect, effect_incomplete, sample_size, FP_complete, IF, groups, Ratio, CONSORT, sample_size_Pub, sample_size_recal, Multicenter, Registration, Design, EP, FC.
(XLSX)
