# Supplementary material for: Reporting of statistical sample size calculations in publications of trials on age-related macular degeneration, glaucoma and cataract
Source: PLoS One. 2021 Jun 4;16(6):e0252640. doi: 10.1371/journal.pone.0252640 (PMC8177464; doi:10.1371/journal.pone.0252640)
Supplement: S1 Table — Recalculated and published sample sizes, difference between recalculated and published sample size, as well as per cent deviations between recalculated and published sample size. (DOCX) [file pone.0252640.s001.docx]

| **Study** | **Calculated sample size** | **Published sample size** | **Absolute Difference** | **Per Cent Deviation** | **Opthalmic Illness** |
| --- | --- | --- | --- | --- | --- |
| **Liu et al. [1]** | **58** | **120** | **62** | **107%** | **AMD** |
| **Rosenfeld et al. [2]** | **99** | **100** | **1** | **1.01%** | **AMD** |
| **Haga et al. [3]** | **28** | **19** | **-9** | **-32.14%** | **AMD** |
| **Ting et al. [4]** | **7** | **13** | **6** | **85.71%** | **Glaucoma** |
| **Miraftabi et al. [5]** | **26** | **25** | **-1** | **-3.85%** | **Glaucoma** |
| **Islamaj et al. [6]** | **52** | **60** | **8** | **15.39%** | **Glaucoma** |
| **Sen et al. [7]** | **23** | **22** | **-1** | **-4.35%** | **Glaucoma** |
| **Kumar et al. [8]** | **45** | **22** | **-23** | **-51.11%** | **Glaucoma** |
| **Cherlerkar et al. [9]** | **40** | **39** | **-1** | **-2.5%** | **Glaucoma** |
| **Das et al. [10]** | **40** | **40** | **0** | **0%** | **Glaucoma** |
| **Quist et al. [11]** | **45** | **45** | **0** | **0%** | **Glaucoma** |
| **Roberts et al. [12]** | **370** | **370** | **0** | **0%** | **Glaucoma** |
| **Zambrowski et al. [13]** | **199** | **200** | **1** | **0.5%** | **Cataract** |
| **Mayro et al. [14]** | **180** | **200** | **20** | **11.11%** | **Cataract** |
| **Ylinen et al. [15]** | **150** | **39** | **-111** | **-74%** | **Cataract** |
| **Gonzales-Salinas et al. [16]** | **85** | **82** | **-3** | **-3.53%** | **Cataract** |
| **Seth et al. [17]** | **64** | **21** | **-43** | **-67.19%** | **Cataract** |
| **Ylinen et al. [18]** | **884** | **57** | **-827** | **-93.55%** | **Cataract** |
| **Choi et al. [19]** | **45** | **22** | **-23** | **-51.11%** | **Cataract** |
| **Manning et al. [20]** | **64** | **63** | **-1** | **-1.56%** | **Cataract** |
| **Ferreira et al. [21]** | **72** | **71** | **-1** | **-1.39%** | **Cataract** |
| **Tzelikis et al. [22]** | **87** | **85** | **-2** | **-2.3%** | **Cataract** |
| **Campa et al. [23]** | **64** | **55** | **-9** | **-14.07%** | **Cataract** |
| **Mendicute et al. [24]** | **27** | **23** | **-4** | **-14.81%** | **Cataract** |

1. Liu K, Song Y, Xu G, Ye J, Wu Z, Liu X, et al. ; PHOENIX Study Group. Conbercept for Treatment of Neovascular Age-related Macular Degeneration: Results of the Randomized Phase 3 PHOENIX Study. Am J Ophthalmol. 2019 Jan;197:156-167. doi: 10.1016/j.ajo.2018.08.026. Epub 2018 Aug 24. PMID: 30148987.
2. Rosenfeld PJ, Dugel PU, Holz FG, Heier JS, Pearlman JA, Novack RL, et al. Emixustat Hydrochloride for Geographic Atrophy Secondary to Age-Related Macular Degeneration: A Randomized Clinical Trial. Ophthalmology. 2018 Oct;125(10):1556-1567. doi: 10.1016/j.ophtha.2018.03.059. Epub 2018 Apr 30. Erratum in: Ophthalmology. 2019 Mar;126(3):471-472. PMID: 29716784.
3. Haga A, Kawaji T, Ideta R, Inomata Y, Tanihara H. Treat-and-extend versus every-other-month regimens with aflibercept in age-related macular degeneration. Acta Ophthalmol. 2018 May;96(3):e393-e398. doi: 10.1111/aos.13607. Epub 2017 Dec 8. PMID: 29220114.
4. Ting JLM, Rudnisky CJ, Damji KF. Prospective randomized controlled trial of phaco-trabectome versus phaco-trabeculectomy in patients with open angle glaucoma. Can J Ophthalmol. 2018 Dec;53(6):588-594. doi: 10.1016/j.jcjo.2018.01.033. Epub 2018 Apr 3. PMID: 30502982.
5. Miraftabi A, Nilforushan N, Darghahi M, Alemzadeh SA, Parsamanesh M, Yadgari M. Effect of subconjunctival Bevacizumab injection on the outcome of Ahmed glaucoma valve implantation: a randomized control trial. Clin Exp Ophthalmol. 2018 Sep;46(7):750-756. doi: 10.1111/ceo.13191. Epub 2018 Apr 6. PMID: 29573081.
6. Islamaj E, Wubbels RJ, de Waard PWT. Primary Baerveldt versus trabeculectomy study after one-year follow-up. Acta Ophthalmol. 2018 Sep;96(6):e740-e746. doi: 10.1111/aos.13658. Epub 2018 Jul 19. PMID: 30022618.
7. Sen M, Midha N, Sidhu T, Angmo D, Sihota R, Dada T. Prospective Randomized Trial Comparing Mitomycin C Combined with Ologen Implant versus Mitomycin C Alone as Adjuvants in Trabeculectomy. Ophthalmol Glaucoma. 2018 Sep-Oct;1(2):88-98. doi: 10.1016/j.ogla.2018.07.003. Epub 2018 Jul 30. PMID: 32677614.
8. Kumar G, Chaurasia RC, Singh SP. Efficacy and Adverse Effects of Topical Latanoprost with Respect to Preservative in Patients of POAG. Journal of Clinical and Diagnostic Research. 2018 Aug, Vol-12(8): FC06-FC09. doi: 10.7860/JCDR/2018/29284.11891.
9. Chelerkar V, Parekh P, Kalyani VKS, Deshpande M, Khandekar R. Comparative Clinical Study of Medically Controlled Nonsevere Chronic Primary Angle-closure Glaucoma with Coexisting Cataract Surgically Managed by Phacoemulsification as against Combined Phacotrabeculectomy. Middle East Afr J Ophthalmol. 2018 Jul-Dec;25(3-4):119-125. doi: 10.4103/meajo.MEAJO_204_17. PMID: 30765948; PMCID: PMC6348945.
10. Das GK, Sahu PK, Kumar S, Biakthangi LVL. Efficacy of Phacotrabeculectomy Alone versus Phacotrabeculectomy Augmented with Autologous Anterior Capsule Implantation Beneath the Sclera Flap. Semin Ophthalmol. 2018;33(2):143-148. doi: 10.1080/08820538.2016.1182558. Epub 2016 Aug 17. PMID: 27533623
11. Quist MS, Brown N, Bicket AK, Herndon LW. The Short-term Effect of Subtenon Sponge Application Versus Subtenon Irrigation of Mitomycin-C on the Outcomes of Trabeculectomy With Ex-PRESS Glaucoma Filtration Device: A Randomized Trial. J Glaucoma. 2018 Feb;27(2):148-156. doi: 10.1097/IJG.0000000000000830. PMID: 29189540.
12. Roberts HW, Wagh VK, Sullivan DL, Hidzheva P, Detesan DI, Heemraz BS, et al. A randomized controlled trial comparing femtosecond laser-assisted cataract surgery versus conventional phacoemulsification surgery. J Cataract Refract Surg. 2019 Jan;45(1):11-20. doi: 10.1016/j.jcrs.2018.08.033. Epub 2018 Nov 7. PMID: 30413333.
13. Zambrowski O, Tavernier E, Souied EH, Desmidt T, Le Gouge A, Bellicaud D, et al. Sleep and mood changes in advanced age after blue-blocking (yellow) intra ocular lens (IOLs) implantation during cataract surgical treatment: a randomized controlled trial. Aging Ment Health. 2018 Oct;22(10):1351-1356. doi: 10.1080/13607863.2017.1348482. Epub 2017 Jul 10. PMID: 28691893.
14. Mayro EL, Pizzi LT, Hark LA, Murchison AP, Wisner D, Koka A, et al. A Proposed Intervention to Decrease Resident-Performed Cataract Surgery Cancellation in a Tertiary Eye Care Center. Am Health Drug Benefits. 2018 Dec;11(9):480-487. PMID: 30746019; PMCID: PMC6322594.
15. Ylinen P, Taipale C, Lindholm JM, Laine I, Holmström E, Tuuminen R. Postoperative management in cataract surgery: nepafenac and preservative-free diclofenac compared. Acta Ophthalmol. 2018 Dec;96(8):853-859. doi: 10.1111/aos.13843. Epub 2018 Oct 3. PMID: 30284393.
16. Gonzalez-Salinas R, Garza-Leon M, Saenz-de-Viteri M, Solis-S JC, Gulias-Cañizo R, Quiroz-Mercado H. Comparison of cumulative dissipated energy delivered by active-fluidic pressure control phacoemulsification system versus gravity-fluidics. Int Ophthalmol. 2018 Oct;38(5):1907-1913. doi: 10.1007/s10792-017-0674-4. Epub 2017 Aug 22. PMID: 28831699.
17. Seth SA, Bansal RK, Ichhpujani P, Seth NG. Comparative evaluation of two toric intraocular lenses for correcting astigmatism in patients undergoing phacoemulsification. Indian J Ophthalmol. 2018 Oct;66(10):1423-1428. doi: 10.4103/ijo.IJO_73_18. PMID: 30249826; PMCID: PMC6173000.
18. Ylinen P, Holmström E, Laine I, Lindholm JM, Tuuminen R. Anti-inflammatory medication following cataract surgery: a randomized trial between preservative-free dexamethasone, diclofenac and their combination. Acta Ophthalmol. 2018 Aug;96(5):486-493. doi: 10.1111/aos.13670. Epub 2018 Jan 25. PMID: 29369527.
19. Choi S, Park SG, Bellan L, Lee HH, Chung SK. Crossover clinical trial of pain relief in cataract surgery. Int Ophthalmol. 2018 Jun;38(3):1027-1033. doi: 10.1007/s10792-017-0554-y. Epub 2017 Jun 20. PMID: 28639089; PMCID: PMC5988792.
20. Manning S, Ugahary LC, Lindstedt EW, Wubbels RJ, van Dissel JT, Jansen JTG, et al. A prospective multicentre randomized placebo-controlled superiority trial in patients with suspected bacterial endophthalmitis after cataract surgery on the adjuvant use of intravitreal dexamethasone to intravitreal antibiotics. Acta Ophthalmol. 2018 Jun;96(4):348-355. doi: 10.1111/aos.13610. Epub 2017 Dec 7. PMID: 29214740.
21. Ferreira TB, Ribeiro FJ, Pinheiro J, Ribeiro P, O'Neill JG. Comparison of Surgically Induced Astigmatism and Morphologic Features Resulting From Femtosecond Laser and Manual Clear Corneal Incisions for Cataract Surgery. J Refract Surg. 2018 May 1;34(5):322-329. doi: 10.3928/1081597X-20180301-01. PMID: 29738588.
22. Tzelikis PF, Morato CS, Neves NT, Hida WT, Alves MR. Intraindividual comparison of nepafenac 0.3% for the prevention of macular edema after phacoemulsification. J Cataract Refract Surg. 2018 Apr;44(4):440-446. doi: 10.1016/j.jcrs.2018.01.026. Epub 2018 Apr 22. PMID: 29685777.
23. Campa C, Salsini G, Perri P. Comparison of the Efficacy of Dexamethasone, Nepafenac, and Bromfenac for Preventing Pseudophakic Cystoid Macular Edema: an Open-label, Prospective, Randomized Controlled Trial. Curr Eye Res. 2018 Mar;43(3):362-367. doi: 10.1080/02713683.2017.1396615. Epub 2017 Nov 9. PMID: 29120255.
24. Mendicute J, Amzallag T, Wang L, Martinez AA. Comparison of incision size and intraocular lens performance after implantation with three preloaded systems and one manual delivery system. Clin Ophthalmol. 2018 Aug 21;12:1495-1503. doi: 10.2147/OPTH.S166776. PMID: 30174410; PMCID: PMC6109656.
