# Supplementary material for: Reporting of statistical sample size calculations in publications of trials on age-related macular degeneration, glaucoma and cataract
Source: PLoS One. 2021 Jun 4;16(6):e0252640. doi: 10.1371/journal.pone.0252640 (PMC8177464; doi:10.1371/journal.pone.0252640)
Supplement: S2 Table — Publication characteristics: CONSORT endorsed by journal, multicentre trial, registered RCT, study design, allocation ratio, published flow chart, primary endpoint, number of groups, impact factor in the year of publication. (DOCX) [file pone.0252640.s002.docx]

| **Characteristic** |  | **All** | **AMD** | **Glaucoma** | **Cataract** |
| --- | --- | --- | --- | --- | --- |
| **CONSORT endorsed**  **by journal** (n = 113) | *Yes* | 19 | 3 | 3 | 13 |
|  | *No* | 94 | 11 | 25 | 58 |
| **Multicentre Trial** (n = 113) | *Yes* | 25 | 5 | 6 | 10 |
|  | *No* | 88 | 9 | 22 | 61 |
| **Listed on a registration platform** (n = 113) | *Yes* | 49 | 8 | 20 | 21 |
|  | *No* | 64 | 6 | 8 | 50 |
| **Studydesign** (n = 113) | *Parallel* | 106 | 13 | 25 | 68 |
|  | *Cross-over* | 7 | 1 | 3 | 3 |
| **Allocation Ratio** (n = 113) | *1:1* | 86 | 10 | 25 | 51 |
|  | *other* | 27 | 4 | 3 | 20 |
| **CONSORT-Flowchart published** (n = 113) | *Yes* | 28 | 6 | 8 | 12 |
|  | *No* | 85 | 8 | 20 | 59 |
| **Primary Endpoint** (n = 113) | *Binary* | 11 | 3 | 6 | 2 |
|  | *continous* | 50 | 9 | 15 | 26 |
|  | *No primary endpoint recorded* | 52 | 2 | 7 | 43 |
| **Number of Groups** (n = 113) | *2* | 92 | 13 | 25 | 54 |
|  | *3* | 18 | - | 3 | 15 |
|  | *4* | 3 | 1 | - | 2 |
| **IF Factor in the year of publication** (n = 113) | *Yes* | 83 | 9 | 19 | 55 |
|  | *No* | 30 | 5 | 9 | 16 |
