# Supplementary material for: Reporting of statistical sample size calculations in publications of trials on age-related macular degeneration, glaucoma and cataract
Source: PLoS One. 2021 Jun 4;16(6):e0252640. doi: 10.1371/journal.pone.0252640 (PMC8177464; doi:10.1371/journal.pone.0252640)
Supplement: S3 Table — Per cent deviations between recalculated and published sample size using the formula (p–b) / b as compared to (b–p) / b. (DOCX) [file pone.0252640.s003.docx]

| (p-b)/b x 100. | $\left( \frac{b-p}{b} \right)\cdot100$ |
| --- | --- |
| 106.8966% | -106.8966% |
| 1.010101% | -1.010101% |
| -32.14286% | 32.14286% |
| 85.71429% | -85.71429% |
| -3.846154% | 3.846154% |
| 15.38462% | -15.38462% |
| -4.347826% | 4.347826% |
| -51.11111% | 51.11111% |
| -2.5% | 2.5% |
| 0% | 0% |
| 0% | 0% |
| 0% | 0% |
| 0.5025126% | -0.5025126% |
| 11.11111% | -11.11111% |
| -74% | 74% |
| -3.529412% | 3.529412% |
| -67.1875% | 67.1875% |
| -93.55204 | 93.55204 |
| -51.11111% | 51.11111% |
| -1.5625% | 1.5625% |
| -1.388889% | 1.388889% |
| -2.298851% | 2.298851% |
| -14.0625% | 14.0625% |
| -14.81481% | 14.81481% |
